# *Coxiella burnetii* is widespread in ticks (Ixodidae) in the Xinjiang areas of China

**DOI:** 10.1186/s12917-020-02538-6

**Published:** 2020-08-28

**Authors:** Jun Ni, Hanliang Lin, Xiaofeng Xu, Qiaoyun Ren, Malike Aizezi, Jin Luo, Yi Luo, Zhan Ma, Ze Chen, Yangchun Tan, Junhui Guo, Wenge Liu, Zhiqiang Qu, Zegong Wu, Jinming Wang, Youquan Li, Guiquan Guan, Jianxun Luo, Hong Yin, Guangyuan Liu

**Affiliations:** 1 State Key Laboratory of Veterinary Etiological Biology, Key Laboratory of Veterinary Parasitology of Gansu Province, Lanzhou Veterinary Research Institute, Chinese Academy of Agricultural Sciences, Xujiaping 1, Lanzhou, Gansu 730046 P. R. China; 2 Animal health supervision institute of Xinjiang Uygur Autonomous Region, Urumqi, Xinjiang 830011 P. R. China; 3 Jiangsu Co-Innovation Center for the Prevention and Control of Important Animal Infectious Disease and Zoonose, Yangzhou University, Yangzhou, Jiangsu 225009 PR China

**Keywords:** *Coxiella burnetii*, Ticks, Ixodidae, Q fever

## Abstract

**Background:**

The gram-negative *Coxiella burnetii* bacterium is the pathogen that causes Q fever. The bacterium is transmitted to animals via ticks, and manure, air, dead infected animals, etc. and can cause infection in domestic animals, wild animals, and humans. Xinjiang, the provincial-level administrative region with the largest land area in China, has many endemic tick species. The infection rate of *C. burnetii* in ticks in Xinjiang border areas has not been studied in detail.

**Results:**

For the current study, 1507 ticks were collected from livestock at 22 sampling sites in ten border regions of the Xinjiang Uygur Autonomous region from 2018 to 2019. *C. burnetii* was detected in 205/348 (58.91%) *Dermacentor nuttalli*; in 110/146 (75.34%) *D. pavlovskyi*; in 66/80 (82.50%) *D. silvarum*; in 15/32 (46.90%) *D. niveus*; in 28/132 (21.21%) *Hyalomma rufipes*; in 24/25 (96.00%) *H. anatolicum*; in 219/312 (70.19%) *H. asiaticum*; in 252/338 (74.56%) *Rhipicephalus sanguineus*; and in 54/92 (58.70%) *Haemaphysalis punctata*. Among these samples, *C. burnetii* was detected in *D. pavlovskyi* for the first time. The infection rate of *Rhipicephalus* was 74.56% (252/338), which was the highest among the four tick genera sampled, whereas the infection rate of *H. anatolicum* was 96% (24/25), which was the highest among the nine tick species sampled. A sequence analysis indicated that 63 16S rRNA sequences could be found in four newly established genotypes: MT498683.1 (*n* = 18), MT498684.1 (*n* = 33), MT498685.1 (*n* = 6), and MT498686.1 (*n* = 6).

**Conclusions:**

This study indicates that MT498684.1 might represent the main *C. burnetii* genotype in the ticks in Xinjiang because it was detected in eight of the tick species studied. The high infection rate of *C. burnetii* detected in the ticks found in domestic animals may indicate a high likelihood of Q fever infection in both domestic animals and humans.

## Background

*Coxiella burnetii*, an obligate gram-negative intracellular bacterium, can cause Q fever disease in humans, survive in the environment for long periods of time, and is often found in the phagolysosome of infected mammalian cells [1, 2]. Given its impact on global public health, it has attracted significant attention for research purposes [3]. In humans, infection with *C. burnetii* causes acute symptoms that include vomiting, headache, pneumonia, fever, and hepatitis, as well as chronic symptoms related to hepatitis, osteomyelitis, endocarditis, and intravascular infection [4, 5]. In animals, infection with *C. burnetii* causes various reproductive problems, including delivery of weak offspring, infertility, postpartum metritis, stillbirth, and abortion [6].

Q fever was first detected in workers at a slaughterhouse in Brisbane, Australia, in 1935 by E.H. Derrick, who named the illness “question fever” [7]. It has also been reported in humans in other countries, including Great Britain, the Netherlands, Spain, Germany, and Switzerland [8–12]. It was first reported to be in China during the 1950s, with *C. burnetii* antibodies being reported in humans from 32 prefectures in 15 provinces of China [13]. Slaughterhouse workers, veterinarians, and farmers are currently at high risk of contracting this relatively rare zoonotic disease [14].

Ticks are widely distributed around the world and are among the most important vectors of human disease, second only to mosquitoes; they are also the main carrier of pathogens in wild animals and livestock [15]. *Coxiella* maintains a symbiotic relationship with ticks and can infect ticks at all tick life stages [16, 17]. It has been isolated from more than 40 species of hard ticks and at least 14 species of soft ticks, indicating the importance of ticks in its transmission [18]. Animals become infected with *C. burnetii* via tick bites, whereas humans become infected mainly via contact with tick excreta, manure, direct contact with birth products, and air [1, 19]. Although the direct transmission of *C. burnetii* to humans through tick bites has not been reported in detail [20], *C. burnetii* has been reported in the milk, birth products, faeces, and urine of the infected animals, to which humans can be exposed and thus become infected with *C. burnetii* by airborne transmission [1].

Xinjiang is the provincial-level administrative region with the largest land area in China. Its boundary is connected with many countries and there are endemic tick species. In the current study, molecular biological methods were used to detect Q fever in tick species collected from the border area of Xinjiang, China, to reveal the species and pathogen-carrying status of the ticks in this region, to analyse the cross-border spread of Q fever. Through this molecular epidemiological survey, the risk of cross-border transmission of tick-borne Q fever and its spread to mainland China was assessed, to provide basic information on the development of effective prevention and control measures for this important tick-borne disease.

## Results

In total, 1507 tick samples were collected from livestock in different regions of the Xinjiang border (Table 1); the samples belonged to one family (Ixodidae), four genera (606 *Dermacentor,* 471 *Hyalomma*, 338 *Rhipicephalus* and 92 *Haemaphysalis*), and ten species (348 *D. nuttalli*, 146 *D. pavlovskyi*, 80 *D. silvarum*, 32 *D. niveus*, 132 *Hy. rufipes*, 2 *Hy. scupense*, 25 *Hy. anatolicum*, 312 *Hy. asiaticum*, 338 *R. sanguineus*, and 92 *Ha. punctata*).

**Table 1:** Detection of *C. burnetii* DNA in ticks according to tick species, origin of ticks

Region	Location	Species	Adjacent farm animals	No. positive/No. examined
Kashgar Prefecture	Kashi	*D. nuttalli*	sheep	97/120 (80.83%)
		*Hy. asiaticum*	sheep	9/40 (22.50%)
		*R. sanguineus*	sheep	65/70 (92.86%)
Ili Kazak Autonomous Prefecture	Gongliu	*Hy. rufipes*	cattle	19/120 (63.33%)
	Yining	*R. sanguineus*	sheep	84/90 (93.33%)
	Xinyuan	*D. silvarum*	sheep	66/80 (82.50%)
	Nilka	*Hy. rufipes*	cattle	9/12 (75.00%)
	Qapqal Xibe	*R. sanguineus*	sheep	35/100 (35.00%)
	Huocheng	*Ha. punctata*	cattle	54/92 (58.70%)
Kizilsu Kirghiz Autonomous Prefecture	Aheqi	*D. pavlovskyi*	sheep	110/146 (75.34%)
	Atushi	*Hy. asiaticum*	cattle	24/25 (96.00%)
Tarbagatay Prefecture	Hoboksar	*D. nuttalli*	sheep	18/23 (78.26%)
	Tacheng	*D. nuttalli*	cattle	9/10 (90.00%)
	Yumin	*D. niveus*	cattle	15/32 (46.88%)
Altay Prefecture	Jeminay	*D. nuttalli*	sheep	6/15 (40.00%)
		*Hy. scupense*	cattle	0/2 (0.00%)
	Qinghe	*D. nuttalli*	sheep	41/46 (89.13%)
	Habahe	*D. nuttalli*	cattle	0/94 (0.00%)
Akesu Prefecture	Akesu	*D. nuttalli*	sheep	34/40 (85.00%)
	Wushi	*Hy. asiaticum*	cattle	28/30 (93.33%)
		*R. sanguineus*	sheep	45/48 (93.75%)
Hotan Prefecture	Pishan	*Hy. asiaticum*	sheep	20/30 (66.67%)
	Karakax	*R. sanguineus*	sheep	23/30 (76.67%)
Hami Prefecture	Barkol Kazak	*Hy. asiaticum*	cattle	0/48 (0.00%)
Changji hui autonomous prefecture	Qitai	*Hy. asiaticum*	cattle	102/104 (98.08%)
BortalaMongolAutonomousPrefecture	Wenquan	*Hy. asiaticum*	cattle	60/60 (100.00%)

In this study, the tick samples were analysed to detect *C. burnetii*. The IS1111 DNA of *C. burnetii* was detected in 973 (973/1507) DNA samples in the following proportions: *D. nuttalli*, 205 (58.91%); *D. pavlovskyi*, 110 (75.34%); *D. silvarum*, 66 (82.50%); *D. niveus*, 15 (46.90%); *H. scupense*, 0 (0.00%); *H. rufipes*, 28 (21%); *H. anatolicum*, 24 (96.00%); *H. asiaticum*, 219 (70.19%); *H. punctata*, 54 (58.70%); and *R. sanguineus* 252 (74.56%). There were significant difference in the infection rate between different species and the reference group (*P* <  0.05) (Table 2). The infection rate of *Dermacentor* and *Rhipicephalus* had statistical significance with the reference group (*P* <  0.05), while the infection rate of *Haemaphysalis* had no statistical significance with the reference group (*P* >  0.05) (Table 2). Three IS1111 positive samples from each sampling site were randomly selected for sequencing the 16S PCR products. After multisequence alignment, the obtained 63 16S rRNA gene sequences formed four sequence clusters: MT498683.1 (*n* = 18) from *R. sanguineus* and *H. asiaticum*; MT498684.1 (*n* = 33) from *D. nuttalli*, *D. pavlovskyi*, *D. silvarum*, *D. niveus*, *H. rufipes*, *H. anatolicum*, *H. asiaticum*, and *R. sanguineus*; MT498685.1 (*n* = 6) from *H. punctata*; and MT498686.1 (*n* = 6) from *D. nuttalli*.

**Table 2:** Tick species and the PCR results of *C. burnetii* from the Xinjiang samples

Family	Genus	Species	No. examined	No. positive (%)	*P*	χ 2	OR (95% CI)	No. examined	No. positive (%)	*P*	χ 2	OR (95% CI)
Ixodidae	*Dermacentor*	*D. nuttalli*	348	205 (58.91)	< 0.05	54.44	0.19 (0.12–3.00)	606	396 (65.35)	< 0.05	6.86	0.72 (0.56–0.92)
		*D. pavlovskyi*	146	110 (75.34)	< 0.05	81.25	0.09 (0.05–0.16)					
		*D. silvarum*	80	66 (82.50)	< 0.05	75.81	0.06 (0.03–0.12)					
		*D. niveus*	32	15 (46.90)	< 0.05	8.77	0.31 (0.14–0.69)					
	*Hyalomma*	*H. scupense*	2	0 (0.00)	–	–	–	471	271 (57.54)			Ref. group
		*H. rufipes*	132	28 (21.21)			Ref. group					
		*H. anatolicum*	25	24 (96.00)	< 0.05	53.07	0.01 (0.00–0.09)					
		*H. asiaticum*	312	219 (70.19)	< 0.05	90.16	0.11 (0.71–0.19)					
	*Haemaphysalis*	*H. punctata*	92	54 (58.70)	< 0.05	32.82	0.19 (0.11–0.34)	92	54 (58.70)	> 0.05	0.04	0.95 (0.61–1.50)
	*Rhipicephalus*	*R. sanguineus*	338	252 (74.56)	< 0.05	112.16	0.09 (0.06–0.15)	338	252 (74.56)	< 0.05	24.94	0.46 (0.34–0.63)
Total			1507	973 (64.57)				1507	973 (64.57)			

**Fig. 1:**
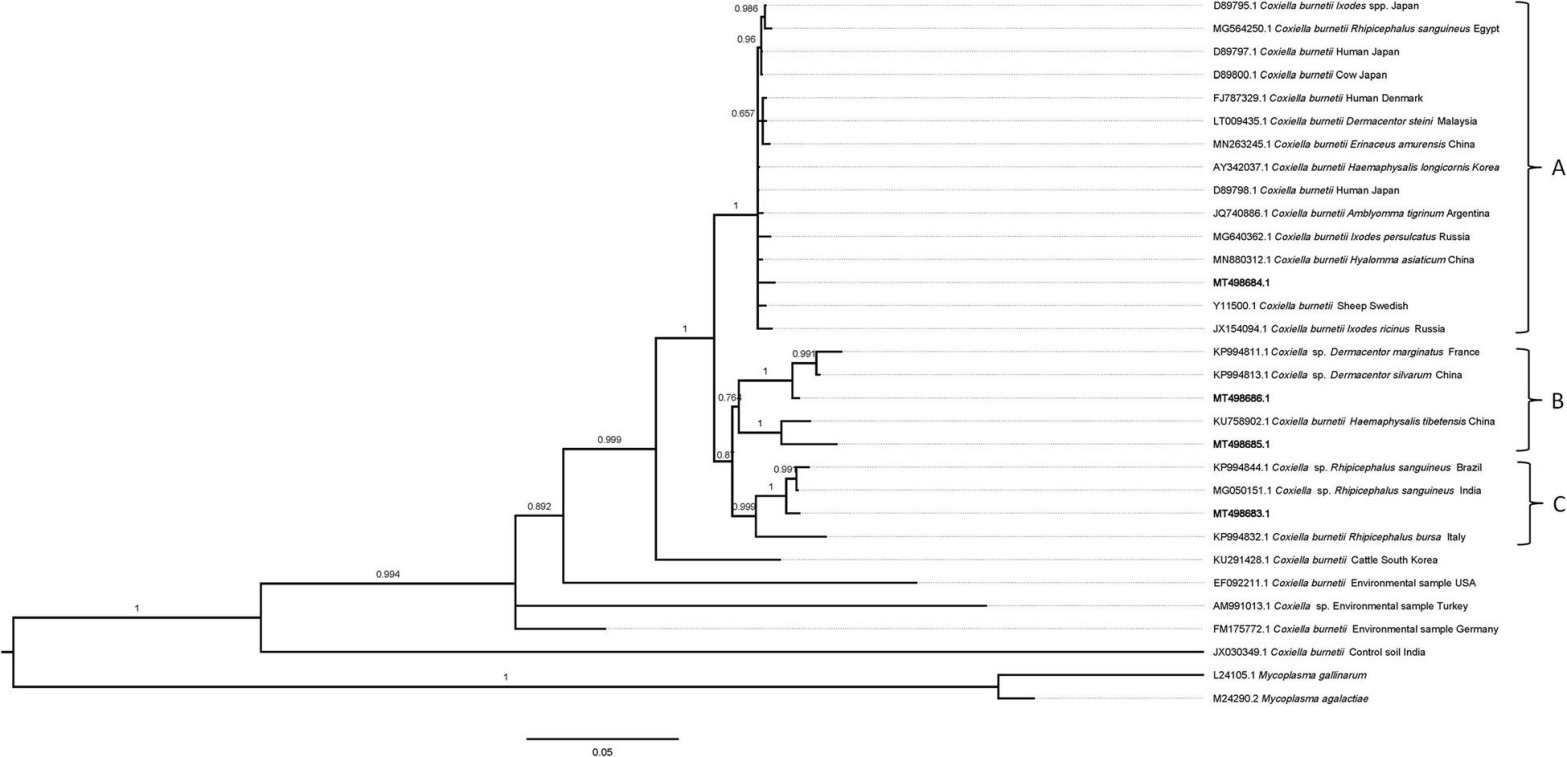
Phylogenetic relationships of the MT498683.1, MT498684.1, MT498685.1, and MT498686.1 genotypes identified in the current study with other *Coxiella* samples by Bayesian inference. Each sequence consists of accession number, host source, and country. The numbers in node represent statistically significant posterior probabilities. The genotypes detected in this study are shown as bold. *Mycoplasma gallinarum* (L24105.1) and *Mycoplasma agalactiae* (M24290.2) were used as the outgroups. The scale bar (0.05) indicating nucleotide substitutions per site

Based on the 16S rRNA gene sequence analysis, the MT498683.1 (*n* = 18) genotype shared 99.3 and 98.5% sequence identity with the *Coxiella* sp. in *R. sanguineus* from India (MG050151.1) and the *C. burnetii* in *Ixodes persulcatus* from the Russia (MG640362.1), respectively; the MT498684.1 (*n* = 33) genotype shared 99.5 and 99.3% sequence identity with the *C. burnetii* in *H. asiaticum* from China (MN880312.1) and the *C. burnetii* in a human (a patient with Q fever endocarditis) from Denmark (FJ787329.1). Respectively, the MT498685.1 (*n* = 6) genotype shared 99.3 and 96.6% sequence identity with the *C. burnetii* in *I. persulcatus* from the Russia (MG640362.1) and the *C. burnetii* in *H. tibetensis* from China (KU758902.1). Finally, the MT498686.1 (*n* = 6) genotype shared 98.4 and 96.6% sequence identity with the *C. burnetii* in *I. ricinus* from Russia (JX154094.1) and the *C. burnetii* in sheep from Swedish (Y11500.1), respectively. As shown by the phylogenetic analysis, the MT498684.1 genotypes belonged to group A, whereas the MT498686.1 and MT498685.1 genotypes belonged to group B, MT498683.1 genotypes belonged to group C (Fig. 1).

## Discussion

As the second largest group of vectors in the world, ticks are hosts to pathogens of a variety of important zoonoses [21–23], such as Forest encephalitis, Q fever, Lyme disease, Spot fever, tularemia, and babesiosis [24, 25]. In recent years, new tick-borne diseases, such as human granulocytic anaplasmosis (HGA), severe fever with thrombocytopenia syndrome (SFTS), and Guertu virus (GTV), have been reported [26, 27]. Ticks transmit pathogens primarily by biting the host [28, 29], but also by aerosol transmission (*C. burnetii*) [5]. Previous reports indicated that ticks are widely distributed in China, with 42 species of ticks from nine genera reported in Xinjiang alone, accounting for more than one-third of the total tick species in China [30–32]. *I. persulcatus, D. nuttalli, H. asiaticum, D. marginatus*, and *D. niveus* are the dominant tick species in Xinjiang [33], and their wide distribution has a significant impact on the development of animal husbandry and public health.

Of the 1507 tick samples collected for the current study, 64.57% (973/1507) contained *C. burnetii* IS1111 DNA. Similarly, previous studies have found a high prevalence of *C. burnetii* in ticks (55.66%) [34] (However, the sample size is too small to accurately reflect the specific infection situation in these areas.); the *R. sanguineus* (60.00%, 3/5) in PTiB, northeastern Spain [35]; the *R. sanguineus* (60.00%, 9/15) in Cyprus [36]. While a lower rate was detected in *D. nuttalli* (12.50%, 7/56) in Gansu, China; the *D. silvarum* (2.79%, 11/394), *D. niveus* (14.75%, 9/61), *H. asiaticum* (22.65%, 41/181) in Xinjiang, China [37–39]; the *H. rufipes* (10.52%, 2/19), *R. sanguineus* (3.44%, 4/116) in Mali [40]; the *H. anatolicum* (13.33%, 2/15) in Cyprus [34]. The differences in infection rate between this study and others may be related to the number of samples, collection location, detection method, and ecological environment. The temperate continental climate provides a good habitat for a wide variety of ticks in Xinjiang. Xinjiang has a great variety of mammalian and avian species, which could serve as hosts of diverse tick species. Therefore, we speculate that climate and invertebrate vector abundance factors may be related to infection.

We did not detect *C. burnetii* in *H. scupense* (0/2; 0.00%), an outcome that may be related to the small sample size (2 samples of *H. scupense*). In addition, the presence of *C. burnetii* in *D. pavlovskyi* was first reported here. The high infection rate of *C. burnetii* in *D. silvarum*, *H. asiaticum*, and *R. sanguineus* seems to be related to the symbiosis and vertical transmission between them [41, 42]. Although there is no related article reporting the existence of symbiotic and vertical propagation of *C. burnetii* in *D. nuttalli*, *D. pavlovskyi*, *D. niveus*, *H. rufipes*, *H. anatolicum*, or *H. punctata*, the findings lead us to suspect that *C. burnetii* has these relationships to these ticks, particularly because some reports have shown that *C. burnetii* has a high infection rate and vertical transmission relationship among some ticks [43–46]. Confirmation of our hypothesis requires more literature support and research to prove; here we are merely stating the supposition. Overall, our results indicate that *C. burnetii* is widespread in the border areas of Xinjiang.

The presence of *C. burnetii* worldwide, including in the environment and in both vertebrate and invertebrate hosts. Phylogenetic analyses indicated that MT498684.1 (from *D. nuttalli*, *D. pavlovskyi*, *D. silvarum*, *D. niveus*, *H. rufipes*, *H. anatolicum*, *H. asiaticum* and *R. sanguineus*) belonged to group A, MT498686.1 (from *D. nuttalli*) and MT498685.1 (from *H. punctata*) belonged to group B, MT498683.1 (from *R. sanguineus* and *H. asiaticum*) belonged to group C (Fig. 1). MT498683.1, MT498686.1, and MT498685.1 genotypes and *Coxiella* sp. from ticks was clustered into a branch (A and B), whereas MT498684.1 genotypes forms a branch with *C. burnetii* from different sources (cows, human, sheep, and ticks) (Fig. 1). The results show that MT498684.1 genotypes have more hosts than MT498683.1, MT498686.1 and MT498685.1 genotypes. MT498684.1 genotypes may have low zoonotic risk, and the diversity of *C. burnetii* hosts indicates that ticks may play an important role in the transmission of *C. burnetii*.

In this study, 16S rRNA assays were used to detect previously unknown genotypes (MT498683.1, MT498684.1, MT498685.1, and MT498686.1) of *C. burnetii* from nine species of tick: *D. nuttalli*, *D. pavlovskyi*, *D. silvarum*, *D. niveus*, *H. rufipes*, *H. anatolicum*, *H. asiaticum*, *R. sanguineus*, and *H. punctata*. However, MT498683.1 was detected only in *R. sanguineus* and *H. asiaticum*, MT498685.1 was detected only in *H. punctata* and MT498686.1 was detected only in *D. nuttalli*, whereas MT498684.1 was detected in *D. nuttalli*, *D. pavlovskyi*, *D. silvarum*, *D. niveus*, *H. rufipes*, *H. anatolicum*, *H. asiaticum*, and *R. sanguineus*. Thus, these results suggest that MT498684.1 is the main genotype in ticks in Xinjiang. More importantly, MT498684.1 showed 99.3 and 99.5% identity with the *C. burnetii* in the human from Denmark (FJ787329.1) and Japan (D89797.1). There have been few reports of the direct transmission of *C. burnetii* to humans through ticks [20]. However, *C. burnetii* has been reported in the milk, birth products, faeces, and urine of infected animals, to which humans can be exposed and thus be infected [1]. At particularly high risk are veterinary personnel, stockyard workers, farmers, hide tannery workers and others who work closely with animals. Sporadic cases of *C. burnetii* in humans are reported each year, although occasionally there are large outbreaks in humans [16, 17]. For example, between 2007 and 2011, a Q fever epidemic occurred in the Netherlands, affecting 4107 people and causing the death of > 50,000 dairy goats. It was thought that most of these infected people developed Q fever by inhaling air in which *C. burnetii* had been released during the birthing season of both goats and sheep (February–May) (http://www.rivm.nl/Onderwerpen/Q/Q_koorts) [9, 47].

In the 1950s, Q fever was first reported in China [48, 49]. The first Chinese strain of *C. burnetii* (Qi Yi) was isolated from a confirmed Q fever patient in 1962 [50]. In the past few decades, *C. burnetii* DNA has been detected in blood samples of human (33.33%, 8/24), goats (25.00%, 4/16), horses (39.50%, 79/200; 22.22%, 4/18), cattle (20.51%, 40/195) from Xinjiang [51, 52], goats from Beijing (4.55%, 2/44) [53], spleen samples of mice in Yunnan (85.19%, 46/54) [54], in ticks from Gansu, Ningxia, Shanxi, Xinjiang, Jilin, Liaoning, Heilongjiang [39, 55, 56].

In summary, this study reports, for the first time, the Q fever infection in ticks in the border areas of Xinjiang, China, indicated that the abundant tick species and high infection rates of *C. burnetii* in the border areas of Xinjiang pose potential threats to domestic animals and humans. Xinjiang, located in northwest China, is bordered by Pakistan, Tajikistan, Mongolia, Kyrgyzstan, India, Afghanistan, Russia and Kazakhstan. Ticks are widely distributed in wild animals and domestic animals across the region, providing increased opportunities for cross-border transmission of *C. burnetii* as global trade intensifies. Thus, there is a need for farmers to adhere to livestock testing and to implement tick control strategies. It is of great significance for public health and safety to reduce the risk of cross-border transmission of the pathogen and its spread to the mainland of China.

## Conclusions

This study confirmed, for the first time, that *C. burnetii* is widely distributed in ticks in Xinjiang, China, indicating that domestic animals and humans in this region may be at risk of being infected with *C. burnetii*. Therefore, there is a need for further research on *C. burnetii* cross-border transmission via ticks. More importantly, there is a need to monitor domestic animals and humans in the region and in tick control in the region to the greatest extent possible to ensure animal and public health safety.

## Methods

### Sample collection and morphological identification of the ticks

**Fig. 2:**
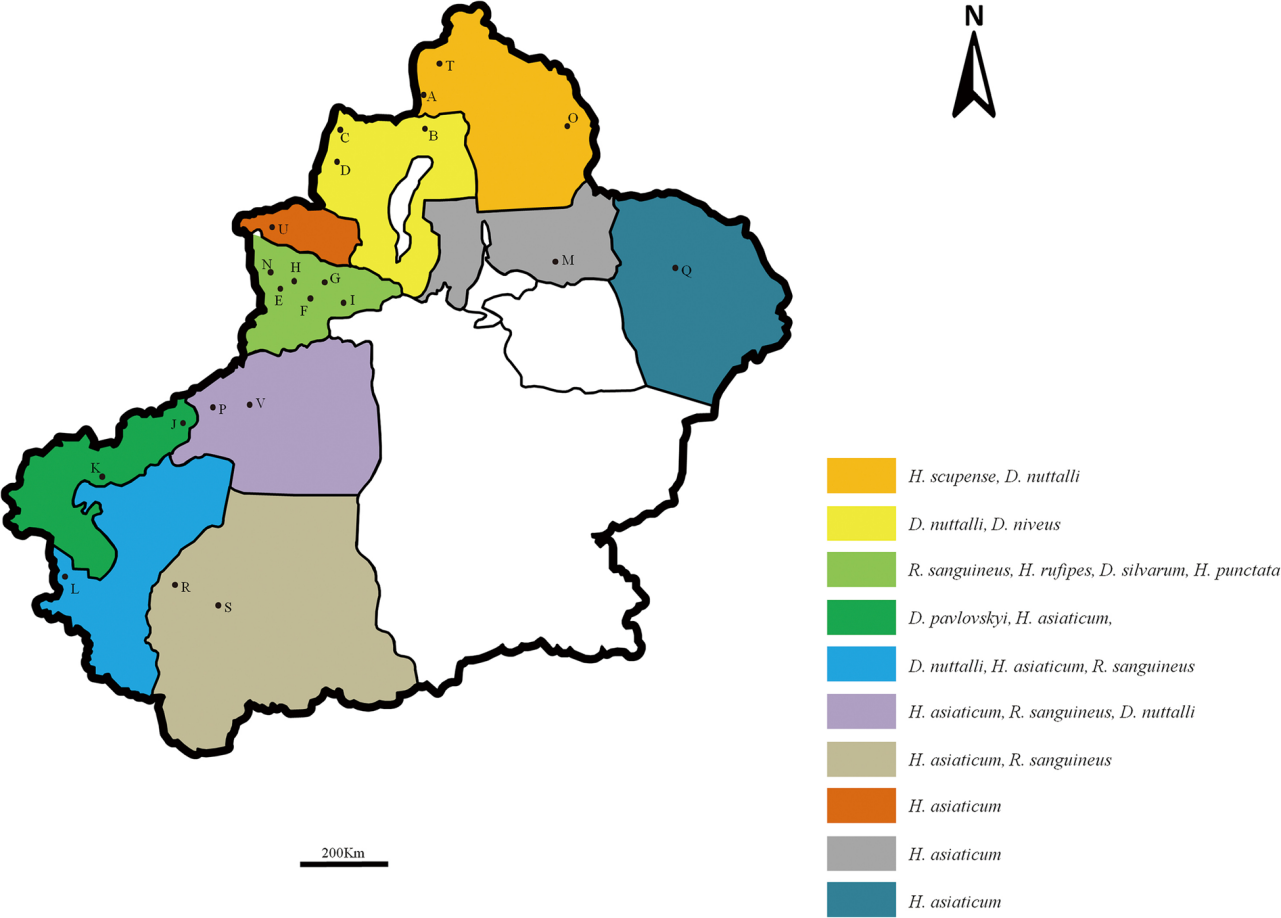
Locations of the sample sites for tick collection in the border areas of Xinjiang (different locations are coded by colour; A–V indicate the sampling points). The map is made by ArcMap 10.2 (https://developers.arcgis.com/)

A total of 1507 ticks were collected from cattle and sheep at 22 sampling sites in ten border regions in the spring of Xinjiang, China from 2018 to 2019 (Fig. 2). The collected samples were stored in 50 mL centrifuge tubes and delivered to the laboratory. The ticks were identified based on morphological criteria following the descriptions provided by Deng GF (1991) [57]. Tick samples were collected with permission from the farmer.

### DNA extraction from tick samples

Tick samples were placed in 50 mL sterile centrifuge tubes and washed individually twice with 75% ethanol, followed by ddH_2_O rinsing until the liquid was clear. For each sample, DNA was extracted using a QIAamp DNA mini kit (Qiagen, Hilden, Germany) according to the manufacturer’s protocol, and the extracted DNA was stored at − 20 °C.

### PCR amplification and sequencing

As described in previous studies [58–61], primers were designed with conserved regions of the *C. burnetii* IS1111 (This is a multi-copy gene encoding a transposase) and 16S rRNA gene sequences (Table 3); the expected product from the *C. burnetii* primers used for IS1111 amplification was 517 bp, and for the 16S rRNA primers, it was 592 bp. The 25 μL of PCR mixture comprised 2 μL of DNA sample, 12.5 μL of DreamTaq Green PCR Master Mix (2×) (Thermo Fisher Scientific, Lithuania, MA, USA), 8.5 μL of ddH_2_O, and 1 μL of 10 μΜ forward primer and 10 μΜ reverse primer (TSINGKE Biotech, Xian, China). A negative control was prepared with double-distilled water, positive control for *C. burnetii* from *H. asiaticum* preserved by our laboratory. Finally, the 25 μL reaction mixture was subjected to PCR under the following conditions: denaturation at 95 °C for 5 min, 95 °C for 30 s, 55 °C for 30 s, and 35 cycles of 72 °C for 1 min, and the final step at 72 °C for 5 min. Next, 5 μL of the PCR products were subjected to 1.5% agarose gel electrophoresis and visualized after being stained with Goldview (Solarbio, Beijing, China); three positive samples (IS1111 gene) from each sampling site were selected for the amplification of *C. burnetii* 16S rRNA sequences. The nucleotide sequences were confirmed by bidirectional sequencing 16S rRNA PCR product in TSINGKE Biotech, China.

**Table 3:** PCR primers used to detect DNA extracted from the ticks taken from Xinjiang

Primers	Target gene	Primer sequence (5′ → 3′)	Annealing temp (°C)	Target fragment (bp)	Reference sequence
F	IS1111	GTGATCTACACGAGACGGGTT	55	517	M80806.1, KT391016.1, KT391020.1, KT391019.1, KT391018.1, KT391017.1, KT954146.1, KT391015.1, KT391014.1, KT391013.1, EU430257.1
R		CGTAATCACCAATCGCTTCGT			
16S-Fw	16S rRNA	TCGGTGGHGAAGAAATTCTC	55	592	KP994776.1, GU797243.1, KP994812.1, KP994826.1, KP994854.1, D89792.1, NR_104916.1, FJ787329.1, HM208383.1, AY342037.1, MH769217.1, MK182891.1
16S-Rv		AGGCACCAARTCATYTCTGACAAG			

### Phylogenetic analysis

Nucleotide sequences were aligned using MAFFT v7 (https://mafft.cbrc.jp/alignment/software/), and use ModelFinder (ModelFinder is implemented in IQ-TREE version 1.6.1.2, http://www.iqtree.org) to calculate the best model (K2P + G4 model was selected based on Bayesian Information Criterion: BIC). Bayesian inference (BI) and Monte Carlo Markov Chain (MCMC) methods were used to construct the phylogenetic tree in PhyloSuite v1.2.2 (https://github.com/dongzhang0725/PhyloSuite/ releases), The number of substitutions (Nst) was set at two, and posterior probability values were calculated by running 2,000,000 generations with four simultaneous tree-building chains. A 50% majority-rule consensus tree was constructed from the final 75% of the trees generated by BI. Analyses were run three times to ensure convergence and insensitivity to priors. Phylogenetic tree were edited in Figtree v1.4.3 (https://github.com/rambaut/figtree/releases).

### Statistical analysis

In this study, Analyses were performed using SPSS Statistics IBM 17 (© IBM Corporation, Somers, New York, USA). Chi-square (χ 2) test was used to conduct statistical analysis on different Genus and species with *C. burnetii* infection.

## Supplementary information

**Additional file 1: Figure S1.** Specificity test results of IS1111 primer.

**Additional file 2: Figure S2.** Sensitivity test results of IS1111 primer.

**Additional file 3: Figure S3.** Specificity test results of 16S rRNA primer.

**Additional file 4: Figure S4.** Sensitivity test results of 16S rRNA primer.

## Data Availability

DNA sequences obtained in this study have been submitted to GenBank database (accession number: MT498683.1-MT498686.1).

## Abbreviations

*C. burnetii*: *Coxiella burnetii*
*D. nuttalli*: *Dermacentor nuttalli*
*D*. *pavlovskyi*: *Dermacentor pavlovskyi*
*D. silvarum*: *Dermacentor silvarum*
*D. niveus*: *Dermacentor niveus*
*H. rufipes*: *Hyalomma rufipes*
*H*. *anatolicum*: *Hyalomma anatolicum*
*H. asiaticum*: *Hyalomma asiaticum*
*R. sanguineus*: *Rhipicephalus sanguineus*
*H. punctata*: *Haemaphysalis punctata*
*H. scupense*: *Hyalomma scupense*
