# Supplementary figures and images for: Coxiella burnetii is widespread in ticks (Ixodidae) in the Xinjiang areas of China
Source: BMC Vet Res. 2020 Aug 28;16:317. doi: 10.1186/s12917-020-02538-6 (PMC7455992; doi:10.1186/s12917-020-02538-6)

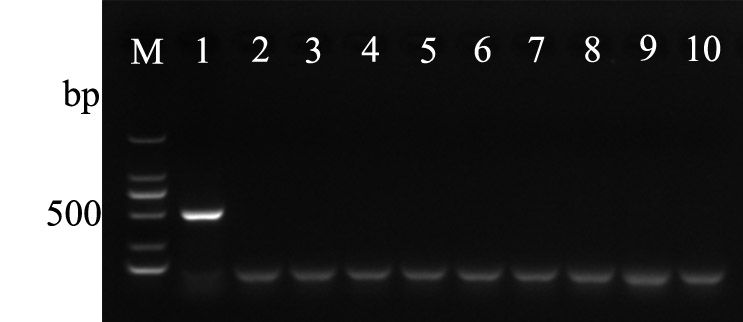

Supplement: Supplementary file 1 — Additional file 1: Figure S1. Specificity test results of IS1111 primer. [file 12917_2020_2538_MOESM1_ESM.jpg]

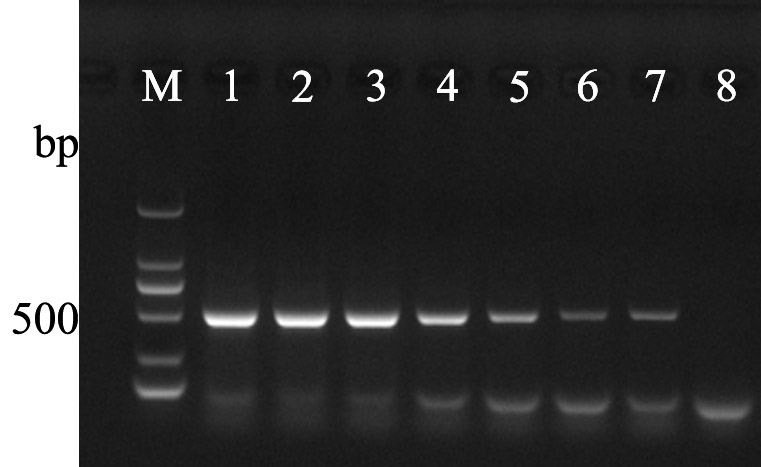

Supplement: Supplementary file 2 — Additional file 2: Figure S2. Sensitivity test results of IS1111 primer. [file 12917_2020_2538_MOESM2_ESM.jpg]

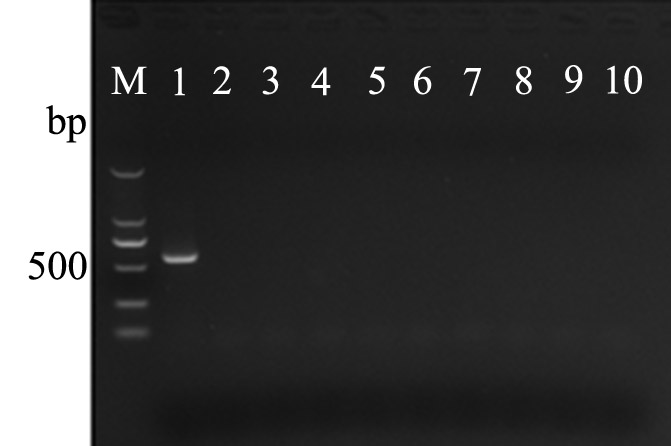

Supplement: Supplementary file 3 — Additional file 3: Figure S3. Specificity test results of 16S rRNA primer. [file 12917_2020_2538_MOESM3_ESM.jpg]

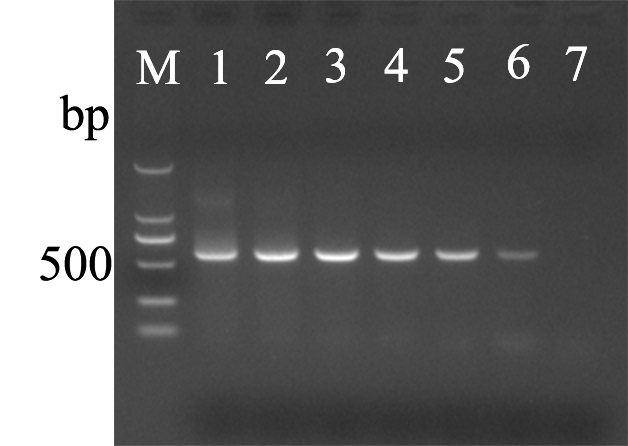

Supplement: Supplementary file 4 — Additional file 4: Figure S4. Sensitivity test results of 16S rRNA primer. [file 12917_2020_2538_MOESM4_ESM.jpg]
